# Supplementary material for: Global prevalence of nosocomial infection: A systematic review and meta-analysis
Source: PLoS One. 2023 Jan 27;18(1):e0274248. doi: 10.1371/journal.pone.0274248 (PMC9882897; doi:10.1371/journal.pone.0274248)
Supplement: S1 File — (PDF) [file pone.0274248.s001.pdf]

First Author/Year	Total Sample	Number of infected (Prevalence)	Country	WHO Region	Year
Duedu,et al 2017	265	232	Ghana	AFRO	2017
Dégbey, et al 2021	384	30	Benin	AFRO	2021
Abdel-Fattah, et al 2005	67	46	Saudi Arabia	EMRO	2005
Abdel-wahab, et al 2013	1373	74	Egypt	EMRO	2013
Abubakar, et al 2020	321	47	Nigeria	AFRO	2020
Abulhasan, et al 2020	562	121	Kuwait	EMRO	2020
Adeleke,et al 2015	231	57	South Africa	AFRO	2015
Afhami, et al 2013	1287	288	Iran	EMRO	2013
Afhami, et al 2019	834	57	Iran	EMRO	2019
Afle, et al 2019	160	104	Benin	AFRO	2019
Ahmadi, et al 2019	717	95	Iran	EMRO	2019
Ahmed, et al 2012	2253	114	Sudan	EMRO	2012
Ahmed,et al 2015	402	84	Tanzania	AFRO	2015
Aiken,et al 2013	1172	93	Kenya	AFRO	2013
Al Luhidan, et al 2019	752	82	Saudi Arabia	EMRO	2019
Al -Tawfiq, et al 2009	57122	588	Saudi Arabia	EMRO	2009
Alebel,et al 2021	270	95	Ethiopia	AFRO	2021
Alemayehu,et al 2014	415	72	Ethiopia	AFRO	2014
Alemu,et al 2012	385	40	Ethiopia	AFRO	2012
Alemu,et al 2020	336	39	Ethiopia	AFRO	2020
Alexopoulos, et al 2011	2180	64	Greece	EURO	2011
Alfayez, et al 2019	248	53	Saudi Arabia	EMRO	2019
Alfouzan, et al 2019	479	163	Kuwait	EMRO	2019
Al-Gasha'a, et al 2020	4013	166	Iraq	EMRO	2020
Al-Hazmi, et al 2015	7923	211	Saudi Arabia	EMRO	2015
Ali,et al 2016	300	42	Ethiopia	AFRO	2016
Aliki, et al 2018	2421	136	Switzerland	EURO	2018
Alnajjar, et al 2020	100	45	United Arab Emirates	EMRO	2020
Alothman, et al 2020	1103	52	Multiple	EMRO	2020
Aloush, et al 2019	108609	55	Multiple	EMRO	2019
Alshammari, et al 2020	432	109	Saudi Arabia	EMRO	2020
Alshamrani, et al 2019	700	194	Saudi Arabia	EMRO	2019
Amiri, et al 2015	14042	1601	Iran	EMRO	2015
Amiyare, et al 2015	18535	1705	Morocco	EMRO	2015
Anggi,et al 2019	54	24	Indonesia	SEARO	2019
Antonioly, et al 2016	1239	119	Italy	EURO	2016
Antonioly, et al 2020	1102	115	Italy	EURO	2020
Archibald,et al 2000	375	233	Malawi	AFRO	2000
Arif, et al 2021	250	100	Pakistan	EMRO	2021
Arnoldo, et al 2018 A	3172	224	Italy	EURO	2018
Arnoldo, et al 2018 B	3253	204	Italy	EURO	2018
Arnoldo, et al 2018 C	2969	164	Italy	EURO	2018
Arnoldo, et al 2018 D	3036	175	Italy	EURO	2018
Asembergiene, et al 2009	1239	169	Lithuania	EURO	2009
Askarian, et al 2004	175	119	Iran	EMRO	2004
Askarian, et al 2011	182	140	Iran	EMRO	2011
Askarian, et al 2013	126	86	Iran	EMRO	2013
Astagneau, et al 2000	236334	18074	France	EURO	2000
Atici, et al 2016 A	86	60	Turkey	EURO	2016
Atici, et al 2016 B	299	63	Turkey	EURO	2016
Atici, et al 2016 C	319	56	Turkey	EURO	2016
Atici, et al 2016 D	303	45	Turkey	EURO	2016
Auriti, et al 2010	1692	217	Italy	EURO	2010
Awad, et al 2020	807	11	Jordan	EMRO	2020
Awoke,et al 2019	261	34	Ethiopia	AFRO	2019
Ayala,et al 2021	382	34	Ethiopia	AFRO	2021
Ayatollahi Mousavi, et al 2018	237	63	Iran	EMRO	2018
Ayed, et al 2019	7235	152	Tunisia	EMRO	2019
Ayele,et al 2018	384	23	Ethiopia	AFRO	2018
Azimi, et al 2011	1053	89	Iran	EMRO	2011
Azzam, et al 2001	7018393	82949	Lebanon	EMRO	2001
Bakullari, et al 2014	79019	2037	United States	AMRO	2014
Balkhy, et al 2006	185	79	Saudi Arabia	EMRO	2006
Baral,et al 2020	426	78	Malawi	AFRO	2020
Barchitta, et al 2020	18852	1042	Italy	EURO	2020
Basiri, et al 2015	434	194	Iran	EMRO	2015
Basu,et al 2017	3128	114	India	SEARO	2017
Bediako-Bowan,et al 2020	4577	438	Ghana	AFRO	2020
Behenke, et al 2013	41539	2110	Germany	EURO	2013
Behnke, et al 2013	41539	2110	Germany	EURO	2013
Behrooozi, et al 2010	96374	993	Iran	EMRO	2010
Behzadnia, et al 2014	200	62	Iran	EMRO	2014

Belete,et al 2019	259	41	Ethiopia	AFRO	2019
Ben Rejeb, et al 2016	1220	55	Tunisia	EMRO	2016
Ben Salem, et al 2011	1500	957	Tunisia	EMRO	2011
Bianco, et al 2018	1283	147	Italy	EURO	2018
Billoro,et al 2019	255	42	Ethiopia	AFRO	2019
Biscione, et al 2009	53501	1772	Brazil	AMRO	2009
Bo, et al 2015	363	147	Italy	EURO	2015
Bolat, et al 2012	1395	156	Turkey	EURO	2012
Boonjaraspinyo,et al 2013	242	90	Thailand	SEARO	2013
Bouzbid, et al 2011	1499	335	France	EURO	2011
Buys,et al 2016	410	339	South Africa	AFRO	2016
Cai, et al 2017	5415	646	China	WPRO	2017
Campos, et al 2001	9322	632	Brazil	AMRO	2001
Carmem Lúcia Pessoa-Silva, et al 2004	4878	1074	Brazil	AMRO	2004
Cevik, et al 2005	190	68	Turkey	EURO	2005
Chabah, et al 2016	811	15	Morocco	EMRO	2016
Chacko,et al 2017	499	76	India	SEARO	2017
Charrier, et al 2014	7825	527	Italy	EURO	2014
Chernet,et al 2020	400	300	Ethiopia	AFRO	2020
Ciofi degli Atti , et al 2012	1506	92	Italy	EURO	2012
Coskun, et al 2008	3249	112	Turkey	EURO	2008
Crivaro, et al 2015	1699	153	Italy	EURO	2015
Daneman, et al 2005	2351	291	Canada	AMRO	2005
Daryapeyma, et al 2016	9894	3294	Sweden	EURO	2016
Dasgupta,et al 2015	242	29	India	SEARO	2015
Davoudi, et 2015	337	57	Iran	EMRO	2015
Davoudi, et al 2014	363	40	Iran	EMRO	2014
Decoster, et al 2012	11182	552	France	EURO	2014
Demeke,et al 2021	281	94	Ethiopia	AFRO	2021
Deorukhkar,et al 2016	13456	93	India	SEARO	2016
Deptu1a , et al 2015	16598	1021	Poland	EURO	2015
Deptuła, et al 2017	945	370	Poland	EURO	2017
Dereje,et al 2017	210	122	Ethiopia	AFRO	2017
Derso,et al 2016	384	121	Ethiopia	AFRO	2016
Dessie,et al 2016	1088	107	Ethiopia	AFRO	2016
Dettenkofer, et al 2001	505	96	Germany	EURO	2001
Dettenkofer, et al 2003	351	169	Germany	EURO	2003
Dhar, et al 2014	80	21	Oman	EMRO	2014
Dima, et al 2007	1739	320	Greece	EURO	2007
Dimitrov, et al 2004	529	28	Kuwait	EMRO	2004
Dimitrov, et al 2005	89	48	Kuwait	EMRO	2005
Ding,et al 2009	1980	531	China	WPRO	2009
Djordjevic, et al 2012	537	89	Serbia	EURO	2012
Douglas, et al 2004	33127	257	Australia	WPRO	2004
Duszynska, et al 2018	2549	540	Poland	EURO	2018
Duszynska, et al 2020	1353	252	Poland	EURO	2020
Efe, et al 2011	621	54	Turkey	EURO	2011
Ehlers,et al 2018	508	253	South Africa	AFRO	2018
Ekrami, et al 2007	6000	135	Iran	EMRO	2007
El Hamzaoui, et al 2020	34556	59	Morocco	EMRO	2020
El Tantawy, et al 2012	377	132	Egypt	EMRO	2012
El-Feky, et al 2016	7454	341	Egypt	EMRO	2016
Ensinck, et al 2018	728	456	Argentina	AMRO	2018
Erbay, et al 2003	434	113	Turkey	EURO	2003
Esen, et al 2004	236	115	Turkey	EURO	2004
Eshetie,et al 2015	442	183	Ethiopia	AFRO	2015
Eshrati, et al 2018	11308	1020	Iran	EMRO	2018
Etyang,et al 2020	68	12	Uganda	AFRO	2020
Eyayu,et al 2021	1240	656	Ethiopia	AFRO	2021
Fabbro-Peray, et al 2007	7086	1914	France	EURO	2007
Fan, et al 2019	27783	856	China	WPRO	2019
Faria, et al 2007	968	163	Albania	EURO	2007
Fekih Hassen, et al 2014	233	94	Tunisia	EMRO	2014
Fenny,et al 2021	357	100	Ghana	AFRO	2021
Fenta,et al 2020	299	50	Ethiopia	AFRO	2020
Ferreira, et al 2020	195	44	Brazil	AMRO	2020
Ferreira,et al 2020	831	263	Mozambique	AFRO	2020
Fisha ,et al 2019	642	64	Ethiopia	AFRO	2019
Fitzpatrick A, et al 2008	3644	198	Ireland	EURO	2008
Fitzpatrick, et al 2008	7541	369	Ireland	EURO	2008
Folgori, et al 2016	14924	335	Italy	EURO	2016
Forson ,et al 2017	50	43	Ghana	AFRO	2017

Gadallah, et al 2014	10075	215	Egypt	EMRO	2014
Gailiene, et al 2012	731	28	Lithuania	EURO	2012
Gajović, et al 2007	2246	180	Serbia	EURO	2007
Ganesh,et al 2019	1597	197	Nepal	SEARO	2019
Gastmeier, et al 2009	652971	2213	Germany	EURO	2009
Geffers, et al 2008	8677	2832	Germany	EURO	2008
Gelaw ,et al 2017	384	26	Ethiopia	AFRO	2017
Gentile, et al 2001	1081	91	Argentina	AMRO	2001
Gentilotti , et al 2020	1040	320	Tanzania	AFRO	2020
Geravandi, et al 2017	171	56	Iran	EMRO	2017
Gerber, et al 2009	1989233	57794	United States	AMRO	2009
Ghassemi, et al 2015	317	115	Iran	EMRO	2015
Ginawi,et al2014	176	46	India	SEARO	2014
Golliot, et al 2001	11254	1118	France	EURO	2001
Graves, et al 2003	5819	553	New Zealand	WPRO	2003
Gregor, et al 2014	21937	189	Czech Republic	EURO	2014
Gugliotta, et al 2020	588	43	Italy	EURO	2020
Guo, et al 2018	986	156	China	WPRO	2018
Gupta, et al 2018	1000	57	Saudi Arabia	EMRO	2018
Gurskis, et al 2009	270	42	Lithuania	EURO	2009
Habyarimana,et al 2021	2910	341	Rwanda	AFRO	2021
Haghighifar, et al 2020	888	116	Iran	EMRO	2020
Hamedi, et al 2014	183	48	Iran	EMRO	2014
Han, et al 2021	480943	3863	China	WPRO	2021
Hassan Al-Wahsh, et 2011	1410	508	Jordan	EMRO	2011
Hassan, et al 2017	2667	232	Egypt	EMRO	2017
Hassan, et al 2020	106	91	Sudan	EMRO	2020
Hassan, R, et al 2017	1731	251	Egypt	EMRO	2017
Hassanzadeh, et al 2009	401	77	Iran	EMRO	2009
Hatachi, et al 2015	426	28	Japan	WPRO	2015
He, et al 2020	325	65	China	WPRO	2020
Hearn, et al 2017	3263	93	Cambodia	WPRO	2017
Heydarpour, et al 2017	312	45	Iran	EMRO	2017
Hormozi, et al 2018	110	19	Iran	EMRO	2018
Hsiue, et al 2010	103	27	Taiwan	WPRO	2010
Hsu, et al 2011	306	74	Taiwan	WPRO	2011
Hugonnet, et al 2007	366	144	Switzerland	EURO	2007
Ilić, et al 2009	764	47	Serbia	EURO	2009
Ilic, et al 2017	2495	153	Serbia	EURO	2017
Iliyasu, et al 2018	8216	518	Nigeria	AFRO	2018
Iwuafor, et al 2016	71	32	Nigeria	AFRO	2016
Jabarpour, et al 2021	3450	324	Iran	EMRO	2021
Jannasch, et al 2015	358	120	Germany	EURO	2015
jaradat, et al 2019	22600	1130	Jordan	EMRO	2019
Jeong, et al 2006	489	148	South Korea	WPRO	2006
Johnson, et al 2021	400	140	Uganda	AFRO	2021
Kahsay, et al 2014	184	73	Ethiopia	AFRO	2014
Kalal,et al 2017	342	62	India	SEARO	2017
Kalayu,et al 2019	649	66	Ethiopia	AFRO	2019
Kaoutar, et al, 2004	1945	518	France	EURO	2004
karaoui, et al 2020	1666	114	Lebanon	EMRO	2020
Katherason, et al 2008	364	128	Malaysia	WPRO	2008
Kaur,et al 2014	1108	174	India	SEARO	2014
Kefale,et al 2020	281	55	Ethiopia	AFRO	2020
Kepenekli, et al, 2015	327	122	Turkey	EURO	2015
Khan, et al, 2001	82	78	Turkey	EURO	2001
Kiani, et al 2009	418	161	Pakistan	EMRO	2009
Kim, et al 2014	617	339	South Korea	WPRO	2014
Kimura, et al 2020	5994054	11823	Japan	WPRO	2020
Kiroro,et al 2018	1086	47	Kenya	AFRO	2018
Kiros,et al 2019	74	11	Ethiopia	AFRO	2019
Kishk, et al 2014	1153	160	Egypt	EMRO	2014
Kivi, et al 2008	93511	3494	Netherlands	EURO	2008
Klavs, et al 2016	5628	358	Slovenia	EURO	2016
Klavs, et al 2019	5743	377	Slovenia	EURO	2019
Kolpa, et al 2018	159028	2184	Poland	EURO	2018
Kołpa, et al, 2018	1847	510	Poland	EURO	2018
König, et al 2021	388	165	Austria	EURO	2021
Krieger, et al 2015	3264	365	Russia	EURO	2015
Krishna,et al 2015	54	43	India	SEARO	2015
Kritsotakis, et al 2008	1832	129	Cyprus	EURO	2008
Kruse, et al 2013	2202	385	Vietnam	WPRO	2013
Kübler, et al 2012	847	206	Poland	EURO	2012

Labi,et al 2019	2107	184	Ghana	AFRO	2019
Lahoorpour, et al 2013	202	79	Iran	EMRO	2013
Lahsaeizadeh, et al 2008	1191	64	Iran	EMRO	2008
Lakhani, et al 2020	293	19	Spain	EURO	2020
Lamarsalle, et al 2013	520715	15520	France	EURO	2013
Lanini, et al 2009	9609	589	Italy	EURO	2009
Lari, et al 2000	505	243	Iran	EMRO	2000
Latha,et al 2019	2249	725	India	SEARO	2019
Le, et al 2021	379	98	China	WPRO	2021
Litwin, et al 2020	3708	742	Poland	EURO	2020
Lizioli, et al 2003	18667	829	Italy	EURO	2003
Lyytikainen, et al 2008	8234	703	Finland	EURO	2008
Madani, et al 2009	458	4	Morocco	EMRO	2008
Magele,et al 2015	984	235	Nairobi	AFRO	2015
Mageto,et al 2020	180	37	Kenya	AFRO	2020
Mahfouz, et al 2010	245	73	Saudi Arabia	EMRO	2010
Mahjoub, et al 2015	7730	103	Tunisia	EMRO	2015
Mama,et al 2014	150	131	Ethiopia	AFRO	2014
Mama,et al 2019	239	81	Ethiopia	AFRO	2019
Mamo,et al 2017	384	36	Ethiopia	AFRO	2017
Manyahi,et al 2014	100	90	Tanzania	AFRO	2014
Mazière, et al 2013	223	38	France	EURO	2013
McKay, et al 2015	543	472	South Africa	AFRO	2015
Medina-Polo, et al 2014	1701	115	Spain	EURO	2014
Melaku,et al 2012	1254	118	Ethiopia	AFRO	2012
Meng, et al 2020	2227	222	China	WPRO	2020
Mengesha,et al 2014	610	123	Ethiopia	AFRO	2014
Metsini, et al 2018	2421	136	Switzerland	EURO	2018
Milošević, et al 2014	52	39	Serbia	EURO	2014
Misal,et al 2017	650	104	India	SEARO	2017
Misganaw,et al 2020	68	16	Ethiopia	AFRO	2020
Mitchel, et al 2016	162503	2821	Australia	WPRO	2016
Mitchell, et al 2019	25578	2447	Canada	AMRO	2019
Mohammed, et al 2014	389	63	Egypt	EMRO	2014
Mohammed, et al 2016	1497	50	Libya	EMRO	2016
Moreno, et al 2006	2172	266	Colombia	AMRO	2006
Mpirimbanyi,et al-2018	175	43	Rwanda	AFRO	2018
Mukagendaneza,et al 2019	294	34	Rwanda	AFRO	2019
Mulu,et al 2012	294	32	Ethiopia	AFRO	2012
Mulu,et al 2015	409	63	Ethiopia	AFRO	2015
Mulu,et al 2017	575	280	Ethiopia	AFRO	2017
Muluye,et al 2013	228	204	Ethiopia	AFRO	2013
Murajda, et al 2010	247	11	Slovakia	EURO	2010
Mushi,et al 2019	250	59	Tanzania	AFRO	2019
Musonda,et al 2020	78	72	Zambia	AFRO	2020
Myat,et al 2020	947	90	Myanmar	SEARO	2020
Naas,et al 2016	8500	303	Madagascar	AFRO	2016
Naderi-Nasab, et al 2007	3686	1738	Iran	EMRO	2007
Nahirya,et al 2008	391	163	Uganda	AFRO	2008
Nangino Gde, et al 2012	974	87	Brazil	AMRO	2012
Ndir, et al 2016	186	110	Senegal	AFRO	2016
Negero,et al 2017	210	159	Ethiopia	AFRO	2017
Ngamchokwathana, etal 2021	156	45	Thailand	SEARO	2021
Ngogo,et al 2020	297	49	Tanzania	AFRO	2020
Ngonzi,et al 2018	4231	84	Uganda	AFRO	2018
Nkurunziza, et al 2019	550	60	Rwanda	AFRO	2019
Nosrati, et al 2010	473	155	Iran	EMRO	2010
Nurain, et al 2015	91	67	Sudan	EMRO	2015
Nwadike, et al 2014	100	14	Nigeria	AFRO	2014
Nwankwo,et al 2014	2920	878	Nigeria	AFRO	2014
O¨ ncu¨ l, et al 2014	658	469	Turkey	EURO	2014
O’Callaghan,et al 2011	333	185	Mozambique	AFRO	2011
Oberdorfer,et al 2009	707	46	Thailand	SEARO	2009
Odoki,et al 2019	267	86	Uganda	AFRO	2019
Ofurum, et al 2010	1409	256	Bahrain	EMRO	2010
Oladeinde,et al 2013	509	357	Nigeria	AFRO	2013
Oladokun,et al 2016	226	22	South Africa	AFRO	2016
Olaechea, et al 2003	6593	1156	Poland	EURO	2003
Olawale,et al 2011	525	118	Nigeria	AFRO	2011
Omar M, et al 2003	5351955	113436	United States	AMRO	2003
Omar, et al 2009	149609	1789	Kuwait	EMRO	2009
Oskouie, et al 2013	31083	470	Iran	EMRO	2013
Parajuli,et al 2017	3088	231	Nepal	SEARO	2017

Pathak,et al 2017	1173	92	India	SEARO	2017
Pellizzer, et al 2008	6352	404	Italy	EURO	2008
Pouladfar, et al 2017	532874	36467	Iran	EMRO	2017
Pourakbari, et al 2012	276	25	Iran	EMRO	2012
Qader, et al 2021	1586	625	Afghanistan	EMRO	2021
Rabasa,et al 2002	194	22	Nigeria	AFRO	2002
Rafa , et al 2021	3028	540	Poland	EURO	2021
Raffaldi, et al 2011	260	25	Italy	EURO	2011
Randrianirina,et al 2010	651	533	Madagascar	AFRO	2010
Rangelova , et al 2020	507	48	Bulgarian	EURO	2020
Rasslan, et al 2012	134	56	Egypt	EMRO	2012
Rejeb, et al 2016	55	22	Tunisia	EMRO	2016
Rezai, et al 2017	4029	509	Iran	EMRO	2017
Richards, et al 2000	498998	15790	United States	AMRO	2000
Ripabelli, et al 2019	224	16	Italy	EURO	2019
Roberts, et al 2000	540	10	Australia	WPRO	2000
Rodríguez-Garcí, et al 2015	82	12	Mexico	AMRO	2015
Roohu,et al 2019	100	24	Nepal	SEARO	2019
Rosenthal, et al 2006	21069	3095	Multiple	EURO	2006
Rosenthal, et al 2020	51	11	Multiple	EMRO	2020
Rosenthal, et al 2020	189	83	Multiple	EMRO	2020
Rosenthal,et al 2004	3319	469	Argentina	AMRO	2004
Rosenthal,et al 2019	2716	266	Multiple	EMRO	2019
Rosmarakis , et al 2007	360	18	Greece	EURO	2007
Rosselló-Urgell, et al 2004	34715	7493	America	EURO	2004
Rotimi, et al 2002	1553	130	Kuwait	EMRO	2002
Rouhi, et al 2018	1545	169	Iran	EMRO	2018
Rozaidi, et al 2001	988	228	Malaysia	WPRO	2001
Russo, et al 2019	6623	2767	Australia	WPRO	2019
Sadaf, et al 2020	238	51	Pakistan	EMRO	2020
Sadeghi, et al 2021	1382	668	Iran	EMRO	2021
Saleem, et al 2018	913	79	Pakistan	EMRO	2018
Salgado Yepez, et al 2017	748	70	Ecuador	AMRO	2017
Salmanov, et al 2019	97340	10986	Ukrain	EURO	2019
Salomao, et al 2008	1031	307	Brazil	AMRO	2008
Sampath Jayawee, et al 2019	818	86	Sri Lanka	SEARO	2019
Sartor, et al 2002	207	7	France	EURO	2002
Sarvikivi, et al 2008	511	66	Finland	EURO	2008
Sax, et al 2001	1928	218	Switzerland	EURO	2001
Scamardo, et al 2020	1265	125	Italy	EURO	2020
See, et al 2013	90515	453	Egypt	EMRO	2013
Segagni Lusignani, et al 2016	4321	268	Austria	EURO	2016
Seid,et al 2018	342	104	Ethiopia	AFRO	2018
Shaikh, et al 2008	333	97	Pakistan	EMRO	2008
Shehab El-Din, et al 2015	192	65	Egypt	EMRO	2015
Shibata, et al 2002	16620	954	Japan	WPRO	2002
Shiferaw,et al 2015	160	95	Ethiopia	AFRO	2015
Shrestha,et al 2019	1962	314	Nepal	SEARO	2019
Siddiqui, et al 2012	2241	69	Pakistan	EMRO	2012
Sime,et al 2020	292	18	Ethiopia	AFRO	2020
Simon, et al 2008	411	181	Multiple	EURO	2008
Sinatra, et al 2013	328	12	Italy	EURO	2013
Singh, et al 2015	28340	1189	India	SEARO	2015
Smyth, et al 2008	75455	5722	Multiple	EURO	2008
Soltani, et al 2012	464	157	Iran	EMRO	2012
Soltani, et al 2016	7500	728	Iran	EMRO	2016
Srun, et al 2013	222	176	Cambodia	WPRO	2013
Starzyk-Łuszcz, et al 2017	164	36	Poland	EURO	2017
Steele,et al 2002	114	80	South Africa	AFRO	2002
Sticchi, et al 2018	3647	376	Italy	EURO	2018
Stoesser, et al 2013	613	65	Combodia	WPRO	2013
Šuljagić, et al 2021	12478	519	Serbia	EURO	2021
Sun, et al 2015	1196	87	China	WPRO	2015
Syaiful,et al 2020	4893	135	Indonesia	SEARO	2020
Tabatabaei, et al 2011	428	31	Iran	EMRO	2011
Taher, et al 2009	306	28	Iran	EMRO	2009
Talaat, et al 2016	474544	2373	Egypt	EMRO	2016
Tang, et al 2020	613	72	China	WPRO	2020
Tarai,et al 2019	969	38	India	SEARO	2019
Tarricone, et al 2020	11461	291	Italy	EURO	2020
Tassew,et al 2020	410	81	Ethiopia	AFRO	2020
Tekin, et al 2013	6932	580	Turkey	EURO	2013
Teklebirhan,et al 2015	305	130	Ethiopia	AFRO	2015

Teweldemedhin,et al 2017	270	180	Ethiopia	AFRO	2017
Tolera,et al 2006	394	29	Ethiopia	AFRO	2006
Tolera,et al 2020	394	29	Ethiopia	AFRO	2020
Tuan, et al 2015	376	25	Vietnam	WPRO	2015
Tukenmez Tigen, et al 2014	1798	309	Turkey	EURO	2014
Valera, et al 2001	104	50	Italy	EURO	2001
Valinteliene, et al 2012	25807	973	Lithuania	EURO	2012
van der Kooi, et al 2010	26937	1934	Netherlands	EURO	2010
Vandael, et al 2020	11800	856	Belgium	EURO	2020
Vergeire-Dalmac, et al 2016	224	63	Philippine	WPRO	2016
Vidaković, et al 2020	148	39	Serbia	EURO	2020
Walaszek, et al 2018	2547	510	Poland	EURO	2018
Wan Hanifah, et al 2000	2367	95	Malaysia	WPRO	2000
Wang, et al 2013	572	246	China	WPRO	2013
Wang, et al 2014	1277	31	China	WPRO	2014
Wang, et al 2016	315209	10117	Taiwan	WPRO	2016
Wang, et al 2018	43146	530	China	WPRO	2018
Wang, et al 2019	23062	972	China	WPRO	2019
Wang, et al 2019	15588	7579	China	WPRO	2019
Wodajo, et al 2017	592	65	Ethiopia	AFRO	2017
Wondimeneh,et al 2014	53	28	Ethiopia	AFRO	2014
Worku, et al 2021	225	22	Ethiopia	AFRO	2021
Wu, et al 2019	12505	600	China	WPRO	2019
Xu, et al 2017	4199	307	China	WPRO	2017
Yallew , et al 2016	908	135	Ethiopia	AFRO	2016
Yapicioglu, et al 2011	596	96	Turkey	EURO	2011
Yin, et al 2018	3588	455	China	WPRO	2018
Zahraei, et al 2012	1879356	10712	Iran	EMRO	2012
Zhang, et al 2015	332	74	China	WPRO	2015
Zhang, et al 2016	4029	147	China	WPRO	2016
Zhang, et al 2017	10000	224	China	WPRO	2017
Zhang, et al 2019	1782	410	China	WPRO	2019
Zhang, et al 2019	633990	23361	China	WPRO	2019
Zhao, et al 2019	60332	1580	China	WPRO	2019
Zhou, et al 2019	51691	1709	China	WPRO	2019
Zingg, et al 2017	16237	726	Multiple	EURO	2017
Zuschneid, et al 2010	21832	262	Germany	EURO	2010
	29159630	555995			
