# Supplementary material for: Global prevalence of nosocomial infection: A systematic review and meta-analysis
Source: PLoS One. 2023 Jan 27;18(1):e0274248. doi: 10.1371/journal.pone.0274248 (PMC9882897; doi:10.1371/journal.pone.0274248)
Supplement: Attachment — Submitted filename: Response to reviewers.docx [file pone.0274248.s004.docx]

Response to reviewers

Editor-in-Chief

**PLOS ONE**

**Art. No**: PONE-D-22-04059

Please find the revised version of our manuscript *“Global, regional, and the national prevalence of nosocomial infection: A systematic review and meta-analysis"*: which we would like to resubmit for publication as a review article in PLOS ONE.

Your comments and those of the reviewers were highly insightful and enabled us to greatly improve the quality of our manuscript. In the following pages there are our point-by-point responses to each of the comments of the reviewers as well as your own comments. Also declaration section revised and completed according to journal guideline.

Revisions in the text are shown using green highlight for additions. We hope that the revisions in the manuscript and our accompanying responses will be sufficient to make our manuscript suitable for publication in PLOS ONE.

We shall look forward to hearing from you at your earliest convenience.

Yours sincerely,

***Corresponding Author:** Ahmad Ghashghaee

**Email:** [ahmad.ghashghaee1996@gmail.com](mailto:ahmad.ghashghaee1996@gmail.com)

| Reviewer Number | Original comments of the reviewer | Reply by the author(s) |
| --- | --- | --- |
| Reviews 1 | This is an interesting review article and the authors have collected a unique dataset. The paper is generally well written and organized. However, in my opinion, there are some shortcomings. Some sentences are not well structured and do not bring out the argument clearly. What is the impact of this review to the public health? What recommendations do you have for stakeholders and policymakers? | Nosocomial infection is one of the most important indicators of hospitals to evaluate the performance of the hospital in terms of patient safety. Our study is done on a global scale so it can be very generalizable and help health decision makers to plan to prevent these types of infections. By reducing nosocomial infections, in addition to improving the patient's safety index, a large amount of the costs incurred by the hospital due to these types of infections will be reduced.  We suggest to decision makers that by focusing on different aspects of nosocomial infections such as age, gender, causes, etc. that we mentioned in this study, comprehensive and practical programs can be used to prevent these infections. |
| Reviews 2 | This is a large meta-analysis evaluating the global prevalence of Hospital-acquired infection (HAI). This is an important topic, and the manuscript includes interesting data on causative pathogens for HAI globally and prevalence rates of HAI. The main issue with this manuscript is the number of studies and heterogenous data. With the number of studies, it is difficult to draw broad conclusions. HAI can have different definitions between studies, countries, and institutions. Several co-variates based on different healthcare systems may be unaccounted for. For example, it is surprising that duration of hospitalization was not an associated factor with HAI as seen in previous studies. I would consider limiting the number of studies and potentially decreasing the time the studies were conducted over. With less studies, the manuscript could include a more direct summary and comparison of data from studies. | We explained in the inclusion and exclusion criteria that we only included studies that provided a clear definition of nosocomial infections. On the other hand, the main purpose of our study was to examine the trend of nosocomial infections in the new century, so if the number of articles is reduced, the main purpose of the article will change.  In relation to heterogeneity, we used subgroup analyzes to solve this problem, which were significantly more efficient. |
| Reviewers Comments | Make the title more concise. National and regional prevalence? | We changed it to : Global prevalence of nosocomial infection: A systematic review and meta-analysis |
| Reviewers Comments | Results: AFR higher in Central Africa than the world. By how much? | We wrote this information in full in the original version of the article, but in the submitted version we had to make corrections due to the word limit.  We added : 0.27 (95% CI, 0.22-0.34) |
| Reviewers Comments | Besides E. coli infected patients…which other microorganisms are you comparing? | We added: such as Coagulase-negative staphylococci, Pseudomonas aeruginosa and Staphylococcus spp. |
| Reviewers Comments | Conclusion. Managers? What is their role? | Hospital managers and health policy makers |
| Reviewers Comments | Rephrase last sentence in first paragraph. Not clear.  Thus, by preventing the prevalence of HAIs instead of allocating hefty sums to the treatment of such infections, managers of healthcare centers can bear much lower costs to manage HAIs. | Therefore, by preventing and reducing nosocomial infections, significant savings will be made in the costs imposed on health centers, the health system and society consequently |
| Reviewers Comments | Which studies?  According to studies, the most prevalent causes of HAIs include urinary tract infections (UTIs), respiratory tract infections (RTIs), circulatory system infections, and surgical site infections. | We add a reference for this statement. |
| Reviewers Comments | Sentence is hanging  Although a number of studies have been conducted on different parts of WHO regions to determine the prevalence rate of HAIs. | Although a number of studies have been conducted on different parts of WHO regions to determine the prevalence rate of HAIs, no systematic review has been conducted globally. |
| Reviewers Comments | You need to show up to which date you acquired the 7031 articles. For future references. | between 2000 and June 2021 |
| Reviewers Comments | Clarify  Increasing rate of HAIs by 0.06% in abstract or 0.6% in results. | 0.06 is correct |
| Reviewers Comments | Italicize scientific names | We changed them |
| Reviewers Comments | What could result in high HAIs in central Africa? | This may be due to the lack of health facilities and resources in this area. The continent is also facing natural crises such as water shortages and droughts, which in turn are increasing nosocomial infections. On the other hand, economic conditions in this region are one of the most important causes of these infections. |
| Reviewers Comments | Why is *S. aureus, P. aeroginosa* and *Klebsiella* the most common HAIs? | These three microorganisms, in addition to being easier to transport than others, have significant resistance to antibiotics. On the other hand, they are more resistant to sterilization and disinfection methods than others. Due to these characteristics, these microorganisms have a higher prevalence rate than others. |
| Reviewers Comments | Are there any recommendations? | We suggest that researchers work on the gaps in our study. For example, conduct studies in countries where no articles on nosocomial infections have been found. On the other hand, studies on the cause and transmission of these infections can greatly help the health system to reduce these types of diseases. |
| Reviewers Comments | Some studies consider COVID 19 as a nosocomial infection. Why did you exclude it? | We did not include Covid-19 disease in nosocomial infections because they have different definitions, and if we included Covid-19 infections in our study, it would falsely increase the prevalence of nosocomial infections in recent years, it would be a significant bias. |
|  |  |  |
