# Supplementary material for: Global prevalence of nosocomial infection: A systematic review and meta-analysis
Source: PLoS One. 2023 Jan 27;18(1):e0274248. doi: 10.1371/journal.pone.0274248 (PMC9882897; doi:10.1371/journal.pone.0274248)
Supplement: Attachment — Submitted filename: renamed_fcd50.docx [file pone.0274248.s005.docx]

**Global prevalence of nosocomial infection: A systematic review and meta-analysis**

**REVIEWERS’ COMMENTS:**

This is a well established review

**Abstract:**

Objectives- Which needs immediate attention. Specific sounds redundant.

**Introduction:**

Remove the irrelevant sentence-It might happen with any kind of infection.

According to studies- Which ones?

Which studies in Europe? Which countries? Any citations?

In the last paragraph, can is subject to speculation. Replace with will

**Methodology**: It is ok

**Results:**

Length of stay- Rewrite the first sentence

Countries based on income- Prevalence in high income countries?

**Discussion-**

Why is certain organisms rated as more common HAIs than others?

**Conclusion-**

Which gaps?
