# Supplementary material for: Global prevalence of nosocomial infection: A systematic review and meta-analysis
Source: PLoS One. 2023 Jan 27;18(1):e0274248. doi: 10.1371/journal.pone.0274248 (PMC9882897; doi:10.1371/journal.pone.0274248)
Supplement: Attachment — Submitted filename: Response to reviewers.docx [file pone.0274248.s006.docx]

Response to reviewers

Editor-in-Chief

**PLOS ONE**

**Art. No**: PONE-D-22-04059

Please find the revised version of our manuscript *“Global, regional, and the national prevalence of nosocomial infection: A systematic review and meta-analysis"*: which we would like to resubmit for publication as a review article in PLOS ONE.

Your comments and those of the reviewers were highly insightful and enabled us to greatly improve the quality of our manuscript. In the following pages there are our point-by-point responses to each of the comments of the reviewers as well as your own comments. Also declaration section revised and completed according to journal guideline.

Revisions in the text are shown using green highlight for additions. We hope that the revisions in the manuscript and our accompanying responses will be sufficient to make our manuscript suitable for publication in PLOS ONE.

We shall look forward to hearing from you at your earliest convenience.

Yours sincerely,

***Corresponding Author:** Ahmad Ghashghaee

**Email:** [ahmad.ghashghaee1996@gmail.com](mailto:ahmad.ghashghaee1996@gmail.com)

| Reviewer Number | Original comments of the reviewer | Reply by the author(s) |
| --- | --- | --- |
| Reviews 1 | Abstract:  Objectives- Which needs immediate attention. Specific sounds redundant. | “ Specific “ was omitted |
| Reviews 1 | Introduction:  Remove the irrelevant sentence-It might happen with any kind of infection.  According to studies- Which ones?  Which studies in Europe? Which countries? Any citations?  In the last paragraph, can is subject to speculation. Replace with will | All the comments were corrected |
| Reviews 1 | Results:  Length of stay- Rewrite the first sentence  Countries based on income- Prevalence in high income countries? | The first sentence was rewrote.  The prevalence of high-income countries was added |
| Reviews 1 | Discussion-  Why is certain organisms rated as more common HAIs than others? | Because these bacteria are more resistant to antibiotics than others. |
| Reviews 1 | Conclusion-  Which gaps? | However, several important gaps were identified such as lack of data in different regions and territories and different areas like the cause of HAIs |
|  |  |  |
