# Supplementary material for: Global prevalence of nosocomial infection: A systematic review and meta-analysis
Source: PLoS One. 2023 Jan 27;18(1):e0274248. doi: 10.1371/journal.pone.0274248 (PMC9882897; doi:10.1371/journal.pone.0274248)
Supplement: Attachment — Submitted filename: Reviewer comments.docx [file pone.0274248.s003.docx]

**Global, regional, and the national prevalence of nosocomial infection: A systematic review and meta-analysis**

**Reviewers Comments**

1. Make the title more concise. National and regional prevalence?
2. ABSTRACT:

Results: AFR higher in Central Africa than the world. By how much?

Besides *E. coli* infected patients…which other microorganisms are you comparing?

Conclusion. Managers? What is their role?

3. MAIN TEXT:

**Introduction:**

- Rephrase last sentence in first paragraph. Not clear.

Thus, by preventing the prevalence of HAIs instead of allocating hefty sums to the treatment of such infections, managers of healthcare centers can bear much lower costs to manage HAIs.

- Which studies?

According to studies, the most prevalent causes of HAIs include urinary tract infections (UTIs), respiratory tract infections (RTIs), circulatory system infections, and surgical site infections.

- Sentence is hanging

Although a number of studies have been conducted on different parts of WHO regions to determine the prevalence rate of HAIs.

**Methods:**

- You need to show up to which date did you acquire the 7031 articles. For future references.

**Results:**

- Clarify

Increasing rate of HAIs by 0.06% in abstract or 0.6% in results.

- Italicize scientific names

**Discussion**

- What could result in high HAIs in central Africa?
- Why is *S. aureus*, *P. aeroginosa* and *Klebsiella* the most common HAIs?

**Conclusion**

- Are there any recommendations?

**Limitations**

- Some studies consider COVID 19 as a nosocomial infection. Why did you exclude it?
